# Maca extracts and estrogen replacement therapy in ovariectomized rats exposed at high altitude

**DOI:** 10.1002/rmb2.12357

**Published:** 2020-11-11

**Authors:** Roberto O. Ybañez‐Julca, Daniel Asunción‐Alvarez, Javier Palacios, Chukwuemeka R. Nwokocha

**Affiliations:** ^1^ Laboratorio de Farmacología Facultad de Farmacia y Bioquímica Universidad Nacional de Trujillo Trujillo Perú; ^2^ Laboratorio de Bioquímica Aplicada Facultad de Ciencias de la Salud Universidad Arturo Prat Iquique Chile; ^3^ Department of Basic Medical Sciences Faculty of Medical Sciences The University of the West Indies Kingston Jamaica

**Keywords:** hormone replacement therapy, hypoxia, *Lepidium meyenii*, ovariectomy, oxidative stress

## Abstract

**Purpose:**

Hormone Replacement Therapy (HRT) and herbal remedies are often used to alleviate menopausal symptoms, but their effects and efficacy at high altitudes presents with several uncertainties. The purpose of this study was to evaluate whether pre‐treatment with maca (*Lepidium meyenii* Walp) improved the tolerance to high altitude on an ovariectomized (OVX) rat model at sea level.

**Method:**

The animals were treated with 17β‐estradiol (200 µg/kg; E2), red and black maca (1.5 g/kg) for 28 days and exposed at high altitude or sea level.

**Result:**

Our findings showed that red and black maca extracts significantly (*P* < .001) reduced the MDA level in OVX rat serum under hypoxia in a similar way to E2. Red and black maca extracts had similar effects with E2, by significantly (*P* < .001) reversing and increasing the ovariectomized induced decrease in cornified endometrial cell number. Under hypoxic conditions, the black maca (*P* < .05) and E2 (*P* < .01) increased the uterine weight in OVX rats. Finally, E2 alone significantly recovered the frequency of the uterine contractile response.

**Conclusion:**

Aqueous extract of *L. meyenii* partially protects the reproductive function in hypobaric hypoxic environment, through the recovery of the cornified endometrial cells and uterine weight in a menopausal model of OVX rats.

## Graphical abstract

Maca partially protects the reproductive function in hypobaric hypoxic environment, through the recovery of the cornified endometrial cells and uterine weight in menopausal model of OVX rats.

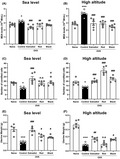

## 1 INTRODUCTION

Around the world, thousands of women use the Hormone Replacement Therapy (HRT) to relieve menopausal symptoms such as hot flashes, genitourinary changes, sexual dysfunction, mood disorders, bone loss, and metabolic changes.^1^ However, the use of HRT in chronic non‐communicable diseases remains controversial.^2^ That means, HRT does not always ameliorate these symptoms in menopausal women because the pathophysiological basis are complex and the root of disease is not only due to hypoestrogenemia.^3^

Clinical evidence for the use of HRT is generally related to people living at sea level, but not in the highlands. Under chronic hypoxia, reproductive age females show lower serum levels of estradiol, progesterone, and prolactin than those living at sea level.^4^ These decreasing female sex hormones are usually associated with late menarche and early menopause.^5^ Research is often focused on the HRT effect during the acclimation process or vasomotor symptoms of the people living at sea level,^6^ but there are very limited studies on the potential interactions between pharmacological therapy and hypobaric hypoxic conditions.

Estrogen depletion has been described to cause a redox imbalance stress,^7^ with an increase in Reactive Oxygen Species (ROS), leading to the enhanced oxidation of polyunsaturated fatty acids in plasma membrane cells.^8^ This phenomenon of lipid peroxidation leads to DNA damage and carbonylation of proteins or enzymes, and produces a great amount of toxic products such as malondialdehyde (MDA).^9^ Furthermore, biometric studies reports that hypobaric hypoxia also generates oxidative stress in humans,^10^, ^11^ through a process involving the reduction of redox mitochondrial potential and an increase in catecholamine concentrations.^12^

Herbal remedies are often used by indigenous persons to manage such ailments, due to its ability to reverse hypoxic‐induced conditions, and stress and cause a recovery of the redox imbalance caused by high altitude.^13^ *Lepidium meyenii* Walp is a plant belonging to the Cruciferae family (Brassicaceae), also called Peruvian Maca; it is cultivated at high altitude of 3800 to 4500 meters above sea level and is rich with strong antioxidant bioactive molecules ^14^ reported to be efficacious in reproductive health, neuroprotection, anticancer, and anti‐hyperplasia.^15^ These includes polyphenols, non‐starch polysaccharides, macamides, glucosinolates, macaenes, and macahydantoins.^16^ Likewise, red and black maca is reported to have positive effects on mood, energy, and chronic mountain sickness ^17^; at high altitude, red maca is reported to generate positive effects on wound healing of male Balb/c mice.^18^

Our study aimed to evaluate whether pre‐treatment with maca protects against high altitude induced changes on a menopausal model at sea level. We compared the uterotrophic activity of *L. meyenii* Walp extracts and the estrogen replacement therapy in ovariectomized rats exposed at high altitude and sea level, so as to ascertain its efficacy, and possible mechanism(s) of action in the management of menopausal symptoms.

## 2 MATERIALS AND METHODS

### 2.1 Animal treatments

**Figure 1:**
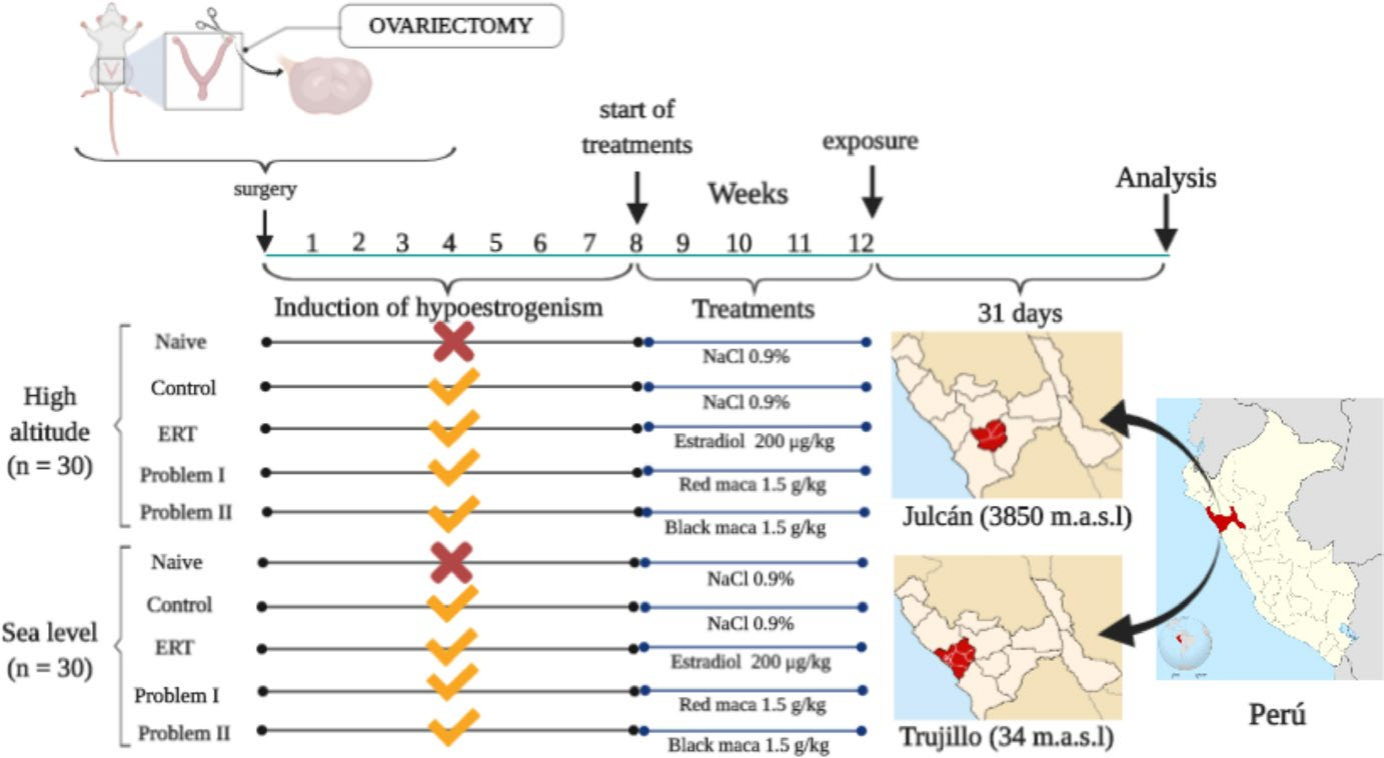
Treatment scheme in the model of ovariectomy‐induced oxidative stress and the influence of high altitude

Sixty female Sprague Dawley rats weighing between 150 and 200 g (2‐3 months old), kept under controlled conditions (12‐h dark/12‐h light cycle, 23‐25 ºC and 50%‐60% humidity) were used for this study. The rats were randomly distributed into five experimental groups (n = 6 per group), as shown in the following scheme (Figure 1). All rats were born at sea level, received standard chow (Molinorte SAC, Trujillo), and were fasted overnight with water ad libitum before the experiments. After 8 weeks, the naive and control groups (OVX) were treated orally once daily with physiological saline. Other OVX groups received various orally administered solutions by gavage, including estradiol valerate (200 µg/kg), red maca (1.5 g/kg), and black maca (1.5 g/kg). The treatment was once per day for 28 continuous days. Then, experiments conducted at high altitude required that thirty animals to be transported by car from Trujillo to Julcán for 31 days. The dose of maca was chosen in accordance with previous studies, which reported 1 to 2 g/kg body weight safe doses of aqueous extracts of maca (red and black) in rats.^19^

### 2.2 Ovariectomy and vaginal cytology

The ovariectomy was performed during a diestrous cycle to keep the consistent lowest levels of sex hormones in rats.^20^ Surgery was performed under anesthesia induced by ketamine (110 mg/kg, i.p.), using standard procedures.^21^ Cells were collected from the vaginal canal were analyzed microscopically and quantified as average number of cells per field of view.^21^

### 2.3 Ex vivo experiment in isolated organ bath: Study of uterine contractility and frequency

Animals were weighed and killed by decapitation. Uterine contractility was evaluated for amplitude and frequency of contraction at basal tone level, and after ex vivo stimulation with oxytocin 0.05 IU and 0.5 IU (non‐cumulative dose) in the isolated organ bath.^21^

### 2.4 Measurement of Malondialdehyde in serum

In a test tube without anticoagulant, 5 mL of blood were collected by cardiac puncture. The obtained supernatant was used for the lipid peroxidation assay based on the method described by Estepa et al.^22^

### 2.5 Statistical Analysis

GraphPad Prism software (San Diego, USA) was used, *n* represents the number of animals studied, and values were expressed as the mean ± standard error of the mean (SEM). For the statistical analysis of the groups, a one‐way ANOVA was used as appropriate, followed by a Bonferroni *post hoc* test. A value *P* of < .05 was considered statistically significant.

## 3 RESULTS

### 3.1 *Lepidium meyenii* aqueous extract and estradiol reduce the MDA levels

**Figure 2:**
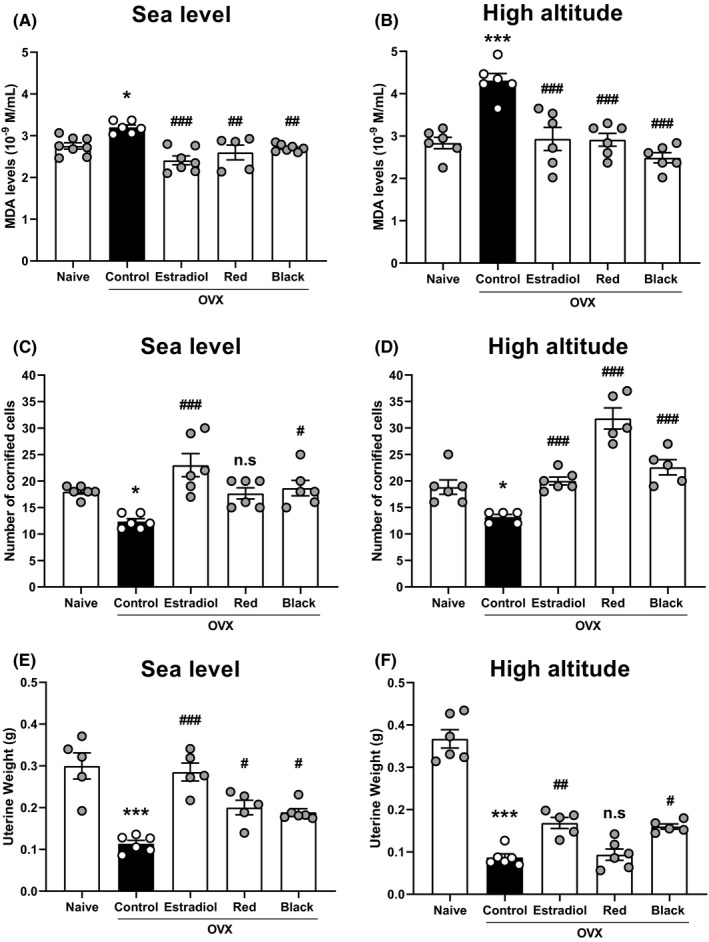
Effect of lyophilized extract of*Lepidium meyenii*Walp (red and black) on malondialdehyde (MDA) levels in serum (A and B). Effect of aqueous extract of*Lepidium meyenii*Walp (red and black ecotype) on the relative number of cornified cells of Sprague Dawley rats (C and D). The number of cornified cells was quantified as average number of cells per field of view. Effect of lyophilized extract of*Lepidium meyenii*Walp (red and black) on uterine weight of Sprague Dawley rats (E and F). Significant differences between groups (mean ± SEM).^#^ *P* < .05,^##^ *P* < .01,^###^ *P* < .001 compared with control group (OVX) and *P < .05, ***P < .001 vs naive group (without ovariectomy). ns = non‐significant statistically compared with control group (OVX)

Since a reduction in estrogen levels due to ovariectomy generates an increase in lipid peroxidation,^7^ we studied whether lipid peroxidation was enhanced in OVX rats exposed at high altitude in the presence or absence of maca and estradiol. Highest levels of lipid peroxidation were found in OVX rats exposed to high altitude (*P* < .001) compared with sea level (intergroup analysis; Figure 2). At sea level, the MDA levels of the control group (OVX rats) increased significantly (*P* < .05) compared with the naive group (without ovariectomy), while, red, and black maca significantly reduced (*P* < .01) MDA levels (3.20 ± 0.17 nmol/ mL control vs 2.60 ± 0.18 nmol/mL red maca, 2.71 ± 0.03 nmol/mL black maca). Intriguingly, the decrease was more significant (*P* < .001) at high altitude than sea level in OVX rats (4.31 ± 0.17 nmol/mL control vs. 2.91 ± 0.15 nmol/mL red maca, 2.49 ± 0.12 nmol/ mL black maca; Figure 2B). Furthermore, estradiol reduced serum MDA levels in both conditions, at sea level (2.41 ± 0.11, *P* < .001; Figure 2A) and at high altitude (2.93 ± 0.27, *P* < .001; Figure 2B) compared with the control.

### 3.2 *Lepidium meyenii* aqueous extract and estradiol increase the number of cornified endometrial cells

It is well known that vaginal smears of rats express the degree of estrogenic effect, by means of an increase in the number of cornified endometrial cells that replace the superficial cells.^23^ Therefore, the effects of *L. meyenii* Walp (red and black maca) extract and estradiol were studied in ovariectomized rats exposed at hypobaric hypoxia.

In both conditions, at sea level and high altitude, the number of cornified endometrial cells of the control group (OVX rats) was significantly reduced (*P* < .05) compared with the naive group (Figure 2C and 2D). At sea level, only black maca significantly increased (*P* < .05) the number of cornified cells in OVX rats (12.33 ± 0.56 control vs 18.7 ± 1.5 black maca; Figure 2C). Otherwise, red and black maca significantly increased (*P* < .001) the number of cornified cells of OVX rats exposed at high altitude (13.2 ± 0.49 control vs 31.8 ± 2.0 red maca, 22.6 ± 1.4 black maca; Figure 2D). Furthermore, although treatment with estradiol increased the number of cornified cells in OVX rats exposed at high altitude (20 ± 0.73, *P* < .05), this effect was more significant at sea level (23 ± 2.18, *P* < .001) when compared with its respective control group. At high altitude, red maca presented a more significant effect compared with estradiol (*P* < .001).

### 3.3 *Lepidium meyenii* aqueous extract and estradiol recover the uterine weight loss in OVX rats

To determine the role of estrogens at the uterine weight, the differences between uterotrophic effect of *L. meyenii* and estradiol in OVX rats exposed at sea level and high altitude were evaluated.

In OVX rats, the uterine weight decreased significantly (*P* < .001) in both conditions, at sea level and at high altitude, compared with the naive group (without ovariectomy; Figure 2E and 2F). Red and black maca increased (*P* < .05) the uterine weight of OVX rats exposed at sea level (0.11 ± 0.008 g control vs 0.20 ± 0.017 g red maca, 0.19 ± 0.009 g black maca; Figure 2E). At high altitude, only black maca increased the uterine weight compared with control (0.087 ± 0.008 g control vs 0.16 ± 0.006 g black maca; Figure 2F). On the other hand, although estradiol increased (*P* < .01) uterine weight at high altitude (0.17 ± 0.013 g), this increase was more significant (*P* < .001) at sea level (0.29 ± 0.021 g) compared with their respective control groups. Furthermore, estradiol increased uterine weight more significantly (*P* < .05) than *L. meyenii* (red and black) at the sea level.

### 3.4 Effect of *Lepidium meyenii* aqueous extract and estradiol on uterine function

**Figure 3:**
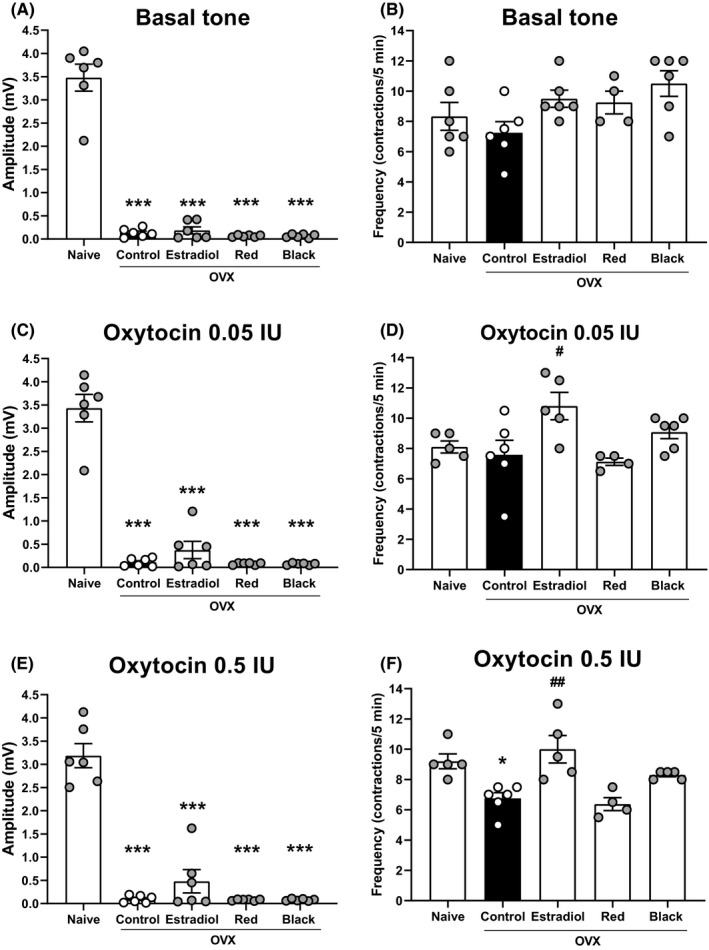
Effect of aqueous extract of*Lepidium meyenii*Walp (red and black ecotype) on uterine function of OVX rats exposed at high altitude (n = 6). Basal tone (A, B), contractile response to 0.05 IU oxytocin (C, D) and 0.5 IU oxytocin (E, F); numbers show the maximum amplitude of contraction (A, C, E) and frequency of contractions (B, D, F). ^#^ *P* < .05, ^##^ *P* < .01 compared with control group (OVX) and *P < .05, ***P < .001 vs naive group

The contractile response of the uterine horns of rats exposed at high altitude was evaluated in order to know whether the uterine function was also altered. Although the ovariectomy blunted the contractile response of uterine horns in all groups in the absence or presence of oxytocin (0.05 and 0.5 IU; Figure 3A, 3C and 3E), the contraction frequency of control group was not decreased compared with naïve group (Figure 3B). The negative effect on the contraction amplitude could not be reversed by the administration of *L. meyenii* or estradiol (Figure 3A, 3C and 3D). But only estradiol caused a significant increase in the contraction frequency compared with the control in the presence of 0.05 IU oxytocin (7.58 ± 0.96 control vs 10.8 ± 0.90 estradiol, *P* < .05; Figure 3D and 0.5 IU oxytocin (6.75 ± 0.38 control vs 10 ± 0.9; *P* < .01 Figure 3F). The contraction frequency was significantly reduced (*P* < .05) by ovariectomy in the control group compared with the naive group (9.2 ± 0.49 naive; Figure 3F). It was shown that the negative effect on the contraction amplitude could not be reversed by the administration of estradiol or *L. meyenii* (0.48 ± 0.25 mV estradiol, 0.079 ± 0.01 mV red maca and 0.08 ± 0.01 mV black maca; Figure 3E).

## 4 DISCUSSION

It is well known that high altitude impairs the female reproductive health.^24^ Lowland women exposed to chronic or intermittent hypoxia have disturbances in the menstrual cycle more than native people from the highlands.^25^ In addition, highland native women presented with an increase in FSH level, early perimenopause,^26^ delayed menarcheal, and advanced menopausal age.^27^ This study compared the effect between estradiol replacement therapy (ERT) and supplementation with *L. meyenii* extracts, a plant rich in phytoestrogens, in animals exposed to hypobaric hypoxia. We found that treatment with *L. meyenii* extract decreased oxidative stress, recovered cornified cell number, and uterine weight in OVX rats exposed to chronic hypoxia, in a similar way with ERT. Uterine function, however, did not recover with treatment. The altitudinal changes are of great importance in the management of menopausal symptoms.

Using ovariectomized rats as our experimental menopause model,^28^ our studies confirmed that ovariectomy of rats increased the lipid peroxidation,^29^ especially in hypoxia‐exposed group. The MDA concentration was also significantly increased when compared with the normoxia (sea level) group. Exposure to hypobaric hypoxia rapidly increases the sympathetic tone, causing an elevation of catecholamines,^30^ like dopamine, with subsequent increases in superoxide anion (•O_2_^‐^) and hydrogen peroxide (H_2_O_2_) by auto‐oxidation.^13^ On the other hand, hypobaric hypoxia also causes a decrease on the cellular available anti‐oxidants in different tissues such as brain,^31^ liver,^32^ lungs, and kidneys.^33^ Hence, hypobaric hypoxia enhances the ovariectomy effects on the imbalance between antioxidant availability and prooxidants (reactive oxygen species; ROS), leading to an enhanced oxidative stress.^12^

To the best of our knowledge, this is the first study that shows how black and red maca extracts reduced the lipid peroxidation in a similar way to estradiol in OVX rats, in both conditions of normoxia and hypoxia. On the one hand, the treatment with 17β‐estradiol is reported to blunt the hypothalamic release of noradrenalin in OVX rats, and an increase in LH,^34^ leading to indirect reduction in prooxidant species.^13^ Thus, estradiol replacement reduces oxidative stress in OVX rats.^35^ On the other hand, chemical composition of *L meyeni* shows polyphenols, glucosinolates, alkamides, and non‐starch polysaccharides which have potent antioxidant activity in vitro ^36^ can also increase the cellular antioxidant status of superoxide dismutase, glutathione peroxidase, and glutathione s‐transferase levels in hereditary hypertriglyceridemic rats,^37^ or even in alcoholic mice.^38^ It has been reported that antioxidant capacity of alkaloids are more relevant than polyphenols metabolites in *L. Meyenii*,^39^ and black maca presents a higher antioxidant activity than red maca.^40^ Therefore, these findings suggest that treatment with maca extracts or estradiol replacement causes a recovery from the imbalance of oxidative stress in the menopausal model of OVX rats exposed to hyperbaric hypoxia.

Chronic hypobaric hypoxia causes an irregular estrous cycle in rats, decreases sex hormone levels, and prolongs diestrous phase.^24^ In OVX rats, the decrease in the number of cornified endometrial cells is directly correlated with an increase in circulating follicle‐stimulating hormone (FSH) and luteinizing hormone (LH), and with lower estrogen levels in plasma.^41^, ^42^ We found that treatment with *L. meyenii* extracts caused a recovery from the decrease in cornified cell number in a similar way to estradiol in OVX rats, in both normoxia and hypoxia conditions. Although the sex hormone levels were not measured in rats, we assume that the reduction in cornified cell number from endometrium by ovariectomy indicate a decrease in estrogen level,^43^ while treatment with maca extracts recovered the effect of estradiol on endometrial cells. This recovery of endometrial cornified cells by maca extracts was more significant in the menopausal model of OVX rats exposed to hypobaric hypoxia when compared with the normoxic animals.

The increase in oxidative stress and impairment of sex hormone levels in OVX rats could be the reason for a significant decrease in uterine weight in the naive group independent of exposure to normoxia or hypoxia conditions. On the contrary, the estradiol replacement countered the decrease in uterine weight in both groups of OVX rats exposed to normoxia and hypoxia. Drop of uterine weight is associated with increase in LH levels and decrease of estrogen in plasma.^44^ The treatment with estradiol of OVX rats increases the proliferation in the uterine endometrium or uterotrophic activity, and this could be mediated by an upregulation of estrogen receptors alpha (ERα) than beta (ERβ) numbers.^45^ Our data are in agreement with a previous study, which showed that aqueous extract of black and red maca attenuated and caused a recovery of the uterine weight loss in OVX mice,^46^ but not with another that used hydroalcoholic extract of black and red maca.^47^ These controversy could be due to the doses and fraction composition employed in different studies causing different physiological effects.^16^ Low doses of maca extracts are reported to increase the release of estradiol and uterotrophic activity in normal rats, while high doses increase the FSH and LH.^48^ Under hypobaric hypoxia condition, only the black maca and estradiol were able to recover the uterine weight in OVX rats. These findings suggest that both extracts of black and red maca may have different constituents and concentrations of compounds and may produce different reproductive effects. Black maca is reportedly rich in polyphenols (quercetin and anthocyanins), polysaccharides, and macamides with antioxidant effects, while red maca is rich in polyphenols and glucosinolates with antiproliferative properties.^16^

Finally, the ovariectomy blunted uterine function in rats exposed to hypobaric hypoxia. Only with estradiol replacement was the frequency significantly recovered of the contractile response, and slightly for the amplitude. These results are supported by previous studies; the estradiol replacement increases the spontaneous contraction of uterus in OVX rats with or without stimulation by oxytocin under normoxia condition.^49^, ^50^ Estradiol replacement upregulates the oxytocin receptors in uterus of rats, while the absence of estrogens in OVX rats causes a downregulation of oxytocin receptors.^51^ The supplementation with maca extracts was not enough to increase the uterine function in similar way with estradiol replacement.

In conclusion, we found that pre‐treatment with aqueous extract of *L. meyenii* partially protects the reproductive function in hypobaric hypoxia environment, through the recovery of cornified cell number and uterine weight in an OVX menopausal rat model, but without recover the uterine function. These findings would be supported by antioxidant and phytoestrogen activity of *L. meyenii*. However, the synergistic effect of its bioactive molecules could also be responsible for the beneficial response to hypoxic exposure, and more research is needed to support the protective effects of the phytoestrogen compounds present in this plant.

## CONFLICT OF INTEREST

Roberto O. Ybañez‐Julca, Daniel Asunción‐Alvarez, Javier Palacios, and Chukwuemeka R. Nwokocha declare that they have no conflict of interest.

## HUMAN AND ANIMAL RIGHTS

This article does not contain any studies with human subjects. All of the institutional and international guidelines for the care and use of laboratory animals (NIH, 2013) were followed.

## APPROVAL BY ETHICS COMMITTEE

The experimental protocols were approved by the Ethics Committee for animal research of the Universidad Nacional de Trujillo (Res. Cons. Univ. No. 0361‐2018/UNT).
